# Mental Health Consequences of COVID-19 Pandemic Period in the European Population: An Institutional Challenge

**DOI:** 10.3390/ijerph19159347

**Published:** 2022-07-30

**Authors:** Nicola Di Fazio, Donato Morena, Giuseppe Delogu, Gianpietro Volonnino, Federico Manetti, Martina Padovano, Matteo Scopetti, Paola Frati, Vittorio Fineschi

**Affiliations:** 1 Department of Anatomical, Histological, Forensic and Orthopaedic Sciences, Sapienza University of Rome, 00185 Roma, Italy; nicola.difazio@uniroma1.it (N.D.F.); donato.morena@uniroma1.it (D.M.); giuseppe.delogu@uniroma1.it (G.D.); gianpietro.volonnino@uniroma1.it (G.V.); federico.manetti@uniroma1.it (F.M.); martina.padovano@uniroma1.it (M.P.); paola.frati@uniroma1.it (P.F.); 2 Department of Medical Surgical Sciences and Translational Medicine, Sapienza University of Rome, 00189 Roma, Italy; matteo.scopetti@uniroma1.it

**Keywords:** COVID-19, pandemic, lockdown, Europe, general population, mental health, psychiatric diseases, psychological burden, health policies

## Abstract

The worldwide spread of SARS-CoV-2 has been responsible for an infectious pandemic, with repercussions on socio-economic aspects and on the physical and mental health of the general population. The present systematic review aimed to evaluate the data belonging to the European framework, analyzing the population by age group. Original articles and reviews on the state of mental health of the general European population have been researched starting from 2021. Initially, a total of 1764 studies were found, among which a total of 75 were selected. Youth were the age group most affected by pandemic consequences on mental health, with emotional and behavioral alterations observed from a third to more than a half of children and adolescents examined. Among both adolescents and adults, the female gender had a higher prevalence of psychopathological symptoms. The main risk factors were poor social support, economic difficulties, and, in particular, unemployment or job changes. Additional individual risk factors were the perception of loneliness, the presence of pre-pandemic mental illness/distress, and some personality traits, such as neuroticism, impulsiveness, and the use of maladaptive coping strategies. Unexpectedly, the elderly maintained good resilience towards change, even if a stress factor was represented by the feeling of loneliness and poor social contact. As regards suicidal behaviors, among adolescents, there was an increase in attempts of 25%, with a greater risk for the female gender. This risk increased also among adults, in association with symptoms of anxiety and depression, and poor socio-environmental conditions. In conclusion, some population groups were found to be at greater risk of psychological burden during pandemic waves, thus representing priority targets for socio-health interventions.

## 1. Introduction

The disease caused by coronavirus SARS-CoV-2, which began in 2019, continues to be a threat to the health of a large part of the global population. SARS-CoV-2 is a highly contagious viral agent, mainly transmitted by air, and causes very variable clinical symptomatology. Infection can either be asymptomatic or lead to cases of severe pneumonia and other life-threatening conditions.

The development of the first cases in China, then in Italy and the rest of the world, led, between 2020 and still today, to public policies of containment of the spread. These were based, especially before the production of vaccines, on social distance, restrictions on movement, quarantines, wearing of protective masks, as well as personal and local sanitation.

Among the most drastic measures, taken especially in the initial stages, there were certainly the so-called ‘lockdowns’, or closures of all productive and commercial activities, schools, universities, and all other social meeting points. Lockdowns involve the obligation to stay at home with a ban on any outside activity except for the rare cases provided for. Subsequently, with the arrival of vaccines, mandatory vaccination measures were introduced for certain population groups [1,2,3].

Although these measures have been considered crucial in reducing the spread of infection, they have, nevertheless, had a significant impact on different areas of society, resulting in an additional burden on the mental health of the population [4].

In such conditions, many sources of stress have accumulated. These ranged from the fear of contracting the infection to that of being responsible for the spread among loved ones. Other stress factors have been represented by prolonged isolation in quarantine because of contracting the infection or being at risk after contact, forced cohabitation—even in unfamiliar environments—changes in living and working habits, as well as a sense of loneliness and uncertainty about the future.

Several international epidemiological studies in the field of mental health, which studied different groups of the population, have reported an increase in anxiety, depression, and stress [5], with worse results for those already presenting a history of mental disorders.

Nonetheless, not all studies have found the same results [6]. This discrepancy could reflect the different precautionary measures taken by countries to cope with the pandemic and the presence of different social risk factors among distinct populations [7].

For example, suicide risk has also been found to be heterogeneous between countries during the pandemic. Actually, while in some countries, such as Japan, there has been an increase in suicide rates, especially among men and young people [8], in other countries, there has been no change in suicide rates compared to previous years [9].

Probably, the messages of solidarity and reciprocity, spread during the periods of greatest social difficulty, and the support initiatives have affected the impact of the pandemic on mental health, but it is also likely that the effects are still far from fully occurring [10].

In this regard, a recent meta-analysis of international data has shown an increased risk of suicide, suicide attempts, and self-injurious behaviors in groups of women and young people [11].

An element of concern is represented by the mental health of children and adolescents and thus the impact of change-related stress at their delicate young age. Before the outbreak of the SARS-CoV-2 pandemic, the world frequency of mental disorders among children and adolescents was estimated at 13.4% [12]; in particular, the prevalence was 6.5% for anxiety disorders, 2.6% for depressive disorders, 3.4% for attention-deficit hyperactivity disorder, and 5.7% for disruptive disorder. The SARS-CoV-2 pandemic has introduced several new sources of stress into the daily lives of children and adolescents.

England’s Mental Health of Children and Young People (MHCYP) follow-up survey [13] showed an increase in the 5- to 16-year-old children’s group probability of having a mental disorder. This increase ranged from 10.8% in 2017 to 16% in 2020. Among the group aged 17–22, this probability rose to 20% overall, with a strong imbalance for female gender (27.2%) compared to male (13.3%).

A review of data from the pandemic early-stage studies [14] showed overall high percentages of anxiety (34.5%), depression (41.7%), irritability (42.3%), and inattention (30.8%) among participants.

Children and adolescents’ feelings of concern, rather than being limited to the fear of contracting the disease, were found to be linked to precarious socio-economic conditions and family stress, lack of parents’ ability to provide support for homework, and difficulty in seeking help and intervention from outside in cases of problems with adults at home [15].

Family stress, often already high in families with children affected by somatic or neuropsychiatric problems, has worsened the well-being of these children, with subsequent increased parents’ concern for their health conditions and the achievement of school goals, in a sort of vicious cycle [16,17].

However, due to the heterogeneity of the samples, the data results were not univocal for children and adolescents [18].

A further vulnerable group is represented by the elderly population. Older people were the most at risk of somatic morbidity and mortality from COVID-19 [19] and also those who suffered from higher levels of loneliness and social isolation than other age groups [20]. For example, before the introduction of vaccines, people older than 65 years had 7.7 times higher COVID-19 death rates than the group aged 55–64 years. In turn, the latter group had an 8.1 times higher COVID-19 mortality rate than individuals younger than 55 years of age [21].

A review of 18 studies conducted by Parlapani et al. [22] pointed out that, despite the greater fear of contracting COVID-19, the elderly population was less burdened than the young and adult population with symptoms of anxiety, depression, psychological, or post-traumatic stress, showing greater well-being. 

Some limitations of these studies were that they had been conducted with a cross-sectional design (only three studies had a longitudinal approach), in populations from developed countries where the impact of psychological crises on the elderly can be mitigated by a particular social organization, and that they used mostly online surveys [23]. These technological means may not be the most significant, inclusive, and effective tools for an epidemiological investigation, especially among the elderly. 

However, regardless of comparison with other population groups, the elderly have experienced an increase in psychological burden, depressive symptoms, anxiety, and more severe stress during the pandemic, with higher levels of loneliness and apathy [24,25,26]. 

The research aimed to collect studies on pandemic-related changes and containment measures implemented by European governments. The focus of interest was set on the European studies in order to reduce the heterogeneity of samples and to highlight some conclusions that could be useful for EU health policies. Thus, the studies were summarized based on the general population, trying to give an overall picture of the various age groups, highlighting the main results regarding mental health changes, and evaluating both risk factors and protective factors. The population groups most at risk of mental suffering were identified, providing the possibility to implement, in the eventuality of new restrictions in the future, health policy strategies of prevention and support.

Although some specific social groups were known to be at greater risk of worsening mental conditions (e.g., people suffering from mental distress [27], people with somatic comorbidities [28], substance users [29], elderly people with dementia [30], caregivers [31], pregnant women [32], refugees and migrants [33], detainees [34], LGBTQ+ [35], health care workers [36], university students [37], etc.), the present review focuses on the impact of pandemic measures on the general population, so the latter was stratified into four main age groups: (1) children, (2) adolescents, (3) adults, and (4) elderly.

In this process, the concept of “vulnerability” has been taken into account as a possibility of exposure to diseases. This concept includes various aspects, such as personal risk factors (biological risk, psychological resilience), social factors (gender, relational, economic, and working conditions), as well as the availability of protective resources for situations at risk [38].

## 2. Materials and Methods

### 2.1. Search Criteria and Critical Appraisal

A systematic literature search through PubMed and a critical appraisal of the collected studies were conducted. The choice of PubMed for the scoping review was determined by the possibility of focusing on medicine and biomedical fields, using a wide number of keywords, searching with various criteria, and accessing readily updated content. Boolean operators, MeSH terms, and free-text terms were used to expand results and not to exclude potentially relevant articles. “English” was applied as a filter, and only articles from 2021 were considered. The last research was carried out on 22 June 2022.

The “Similar articles” sections and the references of the selected articles were consulted to expand the research and to add other articles if of interest.

Search terms were (youth OR child OR children OR adolescents OR young OR women OR girls OR old OR older OR elderly OR unemployed OR unemployment OR psychiatric) AND (coronavirus OR COVID-19 OR COVID) AND (mental health OR distress OR psychological OR stress OR psychiatry) AND Europe.

Preferred Reporting Items for Systematic Review and Meta-Analyses (PRISMA) statement criteria were used in the inclusion of articles in the present systematic review [39,40].

The systematic evaluation implied a quality assessment of the studies based on:

- Methodological framework;
- Adoption of validated evaluation scales, specific for the symptomatologic dimensions investigated;
- Sample size;
- Statistical significance of the results.

Two researchers independently screened for the title and abstract of the papers resulting from the research and selected those of interest for the review.

Disagreements between the two researchers during the articles’ selection phase were resolved through a consensus process. Unpublished and gray literature were not considered. Data were extracted by a single investigator and subsequently verified by another one.

### 2.2. Eligibility Criteria

All the studies, meta-analyses, and review articles on mental health diseases and/or psychological distress during the pandemic time, published since January 2021, were included.

The full texts of the records selected after the first screening were evaluated to retrieve articles that fulfilled the review’s inclusion criteria: (i) original research articles; (ii) focus on the impact of COVID-19 on mental health in the general population, with a selection by age; (iii) use of validated psychometric tools and comprehensive questionnaires to assess specifically the psychological burden of pandemic restrictions. In detail, the focus of the study was addressed on the most common stress-related symptoms and disorders, such as anxiety, depression, post-traumatic stress symptoms, sleep disturbance, loneliness, other than well-being.

Studies adopting only general scales, such as well-being, quality of life, resilience, and stress scales, or that did not measure major psychopathological symptoms were excluded, as well as studies comparing groups exposed and not exposed to COVID-19 or with a focus on specific psychiatric pathologies.

There were no restrictions on study design, that is, cross-sectional or longitudinal, online survey, or face-to-face assessment.

## 3. Results

**Figure 1:**
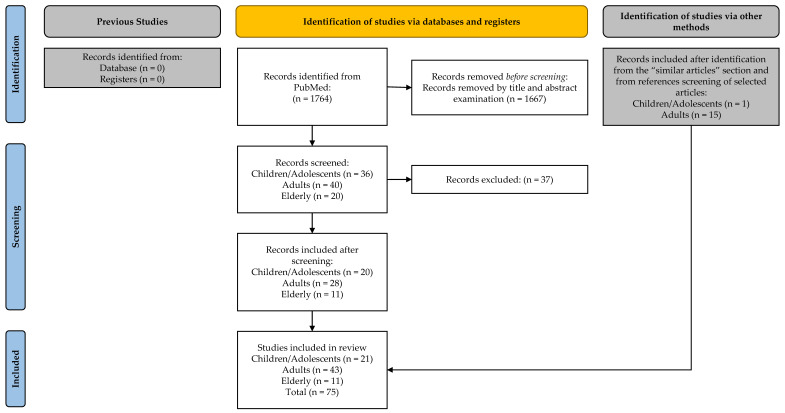
Flowchart of the study selection process.

The application of the search strategies above allowed finding 1764 studies in PubMed. The title and abstract review allowed to select 96 eligible contributions (36 for children/adolescents, 40 for adults, and 20 for the elderly). The full-text review allowed for the selection of 20 eligible contributions for children/adolescents, 28 for adults, and 11 for the elderly. The consultation of “Similar articles” sections and the references of the selected articles permitted to add other papers: 1 for children/adolescents and 15 for adults. At the end of the process, 75 articles were included (Figure 1).

### 3.1. Age Groups

#### 3.1.1. Children

With the onset of the pandemic period and the closure of schools, young children (aged 6–10 years old) experienced an increase in psychological distress, with emotional and behavioral disorders, hyperactivity and distractibility, and a tendency to boredom [41,42,43]. Disturbances of sleep–wake rhythm, poor sleep quality, and difficulty in continuing daily routines occurred in association with hyperactivity and distractibility, and emotional disorders (Table S1).

A large study by Garcia-Adasme et al. [44] found that, during the lockdown, more than half of children under the age of 7 had four or more anxiety-related symptoms and 43.8% of children over the age of 7 had a clinically relevant anxiety disorder.

Some surveys [45] found that, during the lockdown, about a third of the children and adolescents surveyed met the criteria for at least one of the following mental health disorders: attention deficit and hyperactivity disorder, provocative oppositional disorder, depression, anxiety, and problematic use of the internet.

These changes, both emotional and behavioral, were already evident after a short period (8–10 days) from the start of the lockdown [46] and, particularly for hyperactivity, continued to increase progressively.

Notably, younger age emerged as an important risk factor for the development of relational and behavioral symptoms [47].

Other risk factors for hyperactivity and conduct disorders were constituted by having parents or carers with higher levels of psychological distress or having a history of special educational needs (SEN)/neurodevelopmental disorders (ND) [48].

Monnier et al. found that symptoms of hyperactivity/inattention were associated with a long series of additional factors: male gender, access to specialist care before the pandemic and its suspension during school closure, being unschooled or schooled with assistance before the lockdown, tutoring with difficulties or absence of a tutor, and abnormal emotional symptoms.

In turn, the latter have been associated with preterm birth, COVID-19 cases among household members, symptoms of hyperactivity/inattention, and family socioeconomic difficulties.

Another study outlined that a worse psychosocial outcome was associated with socio-family factors such as single-parent families or very large families and negative changes in the working situation for parents or relatives/friends affected by COVID-19. 

#### 3.1.2. Adolescents

About one-third of adolescents had significant emotional disorders related to the stress provoked by the pandemic and related restrictions, exhibiting symptoms of anxiety and depression [49], with a higher prevalence of the former [50] (Table S1).

A symptomatologic interdependence between anxiety and depression was found. During the pandemic, the development of anxiety symptoms increased the risk of depressive symptoms and contributed to a worsening in physical health status [51].

Among adolescents, in general, there was a reduction in all the components of well-being (lifestyle habits, social, and emotional components), as well as a decrease in quality of life and life satisfaction [52,53].

During the pandemic, suicide attempts also increased: data from Catalan registers show, for example, that, among adolescents, they rose by 25% [54].

Regarding mental health status, some studies highlighted that the worst symptomatologic and functional outcome was associated with previous psychopathological or emotional dysregulation problems.

In other studies [55], however, also adolescents with good mental health before the pandemic showed a worsening in many areas: emotional and conduct problems, hyperactivity, and peer relationship problems, with a decrease in their prosocial tendency; interestingly, the opposite occurred for adolescents with worse mental health before the pandemic, for whom there was an improvement in these areas. 

It has been suggested that the lockdown period, at least in the early stages, may have had a beneficial effect on parent-child interactions, with increased supervision and personalized adult support, contributing to improving the mental health of adolescents with a high level of pre-existing mental health problems [56].

Finally, Essau & de la Torre-Luque, clustering the teenagers interviewed into four classes using latent class analysis, ref. [57] found that adolescents with elevated symptoms (23.01% of participants) and with emotional dysregulation (4.79% of participants), that is more than a quarter of the total, had a worse outcome during the lockdown, experiencing more stress, conflict, and loneliness, and lower levels of perceived social support. Adolescents with high levels of emotional dysregulation also tended to consume more alcohol and had a worse family financial situation. 

Analysis of psychological problems and behavioral traits is crucial for research in this field as, for example, general studies on adolescents have found a reduction in cigarette smoking, e-cigarette use, and alcohol intoxication. This evidence is probably linked to a reduction in general social events [58].

Some studies, specifically conducted, identified stressful and risk factors related to psychopathological worsening among adolescents. The main stressors were the interruption of social life, subjectively important activities, and sporting activities. Fear of infection emerged as a minor stressing factor.

Among the risk factors concerning both the development of psychological symptoms and relational problems, several studies identified the following: single-parent families or the absence of brothers/sisters, low socioeconomic level, and unfavorable milieu.

From a gender perspective, a greater perception of stress by women has emerged. In most studies, the female gender was associated with an increased risk of anxiety and depression [59], with a very strong correlation confirmed on a large scale [60]. Suicide attempts also increased significantly more among girls, from three to about five times that of boys (from 99.2 to 146.8/100.000 vs. 32.1–32.3/100.000). 

Inversely, some studies identified protective factors for mental health. For example, it has been found that adolescents are very likely to request forms of help and psychological support through digital platforms.

Finally, some positive psychological elements emerged from some studies. There have been neurocognitive improvements linked to prolonged stay at home, probably due to a reduction in school-related burnout. A rapid improvement in symptomatology was also noted following the suspension of the confinement measures, reflecting the recovery ability of the youngsters.

In this regard, a longitudinal study conducted by Meda et al. [61] through interviews with students between 18 and 30 years old, before, during, and after the first lockdown between October 2019 and June 2020, highlighted how they reported worse depressive symptoms on average during the lockdown months compared to the previous six months, with the prospect that about 6% of them could develop more severe depressive symptoms. However, the symptoms tended to improve rapidly after the lockdown ended.

#### 3.1.3. Adults 

Prevalence data for psychopathological symptoms during the lockdown show a significant increase in the adult population concerning anxiety, depression, somatization, post-traumatic stress, and loneliness [62,63,64,65,66,67] (Table S2).

Specifically, anxiety and depressive symptoms increased both in terms of prevalence (mostly over 30% of respondents) and severity [68].

In addition to an altered psychopathological status, several cognitive tasks, such as attention, temporal orientation, and executive functions, were also affected by confinement. Other symptoms presented by respondents during the lockdown were abnormal sleep, appetite changes, reduced libido, and health anxiety.

Furthermore, the presence of symptoms of anxiety and depression, together with unfavorable socio-environmental conditions, contributed to an increase in suicide risk and substance use [69].

In the European studies analyzed, the female gender was found to be significantly more at risk than the male for the development of stress and anxiety, depression [70,71,72,73,74,75,76,77,78], and post-traumatic symptoms [79,80,81]. The evolution of symptoms over time was also worse for women [82].

Muro et al. [83] showed that increased neuroticism and baseline levels of anxiety and depression were the aspects of personality most at risk for women’s mental health.

Young age emerged as another risk factor for the development of anxious and depressive symptoms [84,85,86,87], although young people were less afraid of contracting the infection and dying compared to older people [88]. Analysis of the various studies also revealed a wide range of socio-demographic and psychological risk factors related to the worsening of stress and psychopathological status.

Among the socio-demographic risk factors the studies found were:

- Low schooling;
- Unemployment and, in general, work difficulties or changes in job or working methods [89,90];
- Low socioeconomic level and unfavorable environmental context [91] regarding the socioeconomic level; however, a large study of the Norwegian population did not show substantial differences between the levels of depression in the population, which were homogeneously increased regardless of socioeconomic status;
- Single, unmarried, or divorced status;
- Residence in urban areas;
- Residence in a European country [92];
- Medical history of somatic health problems or SARS-CoV-2 infection.

Among the psychological risk factors were:

- High perception of loneliness, which revealed to be in a mutual relationship with depressive symptoms [93] and with post-traumatic stress. Moreover, high levels of loneliness, both before and during the pandemic, contributed to the persistence of the other psychopathological symptoms [94];
- Family distress, such as relational difficulties at home, inability to stay alone, loneliness or a feeling of being abandoned, and increased burden of daily duties, were associated with depressive symptoms and generalized anxiety;
- Poorer skill in emotion regulation through cognitive reappraisal;
- Higher level of irritability and impulsivity;
- Emotion regulation difficulties, with an effect on depressive symptoms;
- Low resilience and use of coping strategies of denial and self-blame, particularly related to post-traumatic stress symptoms;
- Fear of contracting COVID-19 [95], albeit with a smaller-size effect than generalized anxiety symptoms;
- A low level of spiritual well-being.

An element that deserves particular attention is represented by the history of mental distress: although not confirmed by all studies, in most of those analyzed, the presence of a vulnerability or history of mental distress was a frequent predisposing factor for the development and increase in symptoms of anxiety and depression, and for worse quality of life [96,97]. 

People with a history of poor mental health also tended to maintain a higher level of severity stably over time and less related to external factors [98]. 

Another relevant element is that, while some risk factors differed according to the countries examined, others remained almost constant, such as being single, students, or parents in young adulthood [99].

Protective factors were also identified, both for a lower severity of symptoms and for a more rapid psychopathological recovery. For example, the following emerged:

- Higher degree of psychopathological well-being and healthy habits before lockdown;
- Improvement in family relations and economic status, having more people living in the household, and the presence of children;
- The ability to regulate emotions and to cope with the new situation caused by the pandemic, finding positive activities (social, work, entertainment) even in conditions of domestic confinement, as well as an extroverted character style and engagement in routines and physical activity.

As regards the temporal evolution of psychological disorders, a decline in mental health status was observed very rapidly after the introduction of restrictions, already in the first days of lockdown.

With the repetition of pandemic waves and related restrictive measures, increased psychological vulnerability emerged, with a progressive increase in the percentage of people suffering from at least one mental disease.

It was also found that, for each month spent during the most difficult phases of the pandemic, there was an approximate 6% increase in the likelihood of falling into moderate to severe depression, anxiety, and stress-related illnesses.

Inversely, in a longitudinal study conducted between March and May 2020 in the UK, a declining trend in the prevalence of anxiety symptoms over time has been registered, while the values of depressive symptoms remained constantly high and the suicidal ideation increased (from 8.2% during the first wave to 9.8% during the third). Such results appeared to be similar for both genders.

Prospective studies conducted after the end of the lockdown provided unequivocal data regarding the evolution of patients’ symptoms. People with depressive disorders experienced greater difficulties in recovering, and, while anxiety and depressive symptoms began to decrease at the end of restrictions, high levels of psychological distress tended to persist [100,101]. Other studies confirmed that psychopathological symptoms remained in the days following the end of the lockdown, along with changes in daily habits (nutrition, sport, screen time).

The likelihood of suffering from depressive symptoms in post-lockdown was almost doubled in cases of high loneliness scores, whereas protective factors were older age and higher resilience. Suicidal ideation was mitigated by the perception of social support and resilience [102]. 

Again, groups at higher risk for the persistence of poor outcomes were women, youngsters, and those who used emotional suppression and internalization as fundamental coping strategies, as well as those presenting pre-existing mental illness.

#### 3.1.4. Elderly 

Although older-age populations were considered less affected in studies on general population mental health during the pandemic and have been protected by specific regulatory measures [103], they also experienced high levels of mental distress (Table S3). 

During the restriction phase, more than half of women and one-third of men over 60 years old presented a form of distress [104], with an emotional response of avoidance or depression. Similar results were also found in other studies with numerically smaller groups [105].

In a longitudinal population study conducted in the UK [106], comprising 5146 people aged over 50 years, there was a steady increase in symptoms of anxiety and depression between June and December 2020. The worst outcomes were associated with female sex and non-partnered status.

Assessment of post-traumatic stress symptoms at the end of the first lockdown period showed the presence of mild symptomatology in 72% of respondents, moderate in 16%, and severe in 4% [107]. Fear of infection was a risk factor for the psychological distress in the elderly, although this was counteracted by their resilience.

During the lockdown, the most exposed to the risks were, once again, isolated people with fewer social contacts, including non-physical ones [108]. Loneliness represented a decisive mediating variable between social restrictions and symptoms of anxiety and depression [109], markedly in a longitudinal perspective, during repeated waves and restrictions [110]. Levels of loneliness were seen to have different patterns between gender and ages [111]. In women, they have a U-shaped trend across ages, with the highest values below 45 years and above 74 years. In men, conversely, the levels of loneliness are highest in young people between 18 and 24 years and gradually reduce according to age. 

A review of data including more than forty thousand people over 50 years, from 17 European countries, has established that lockdown policies have contributed, in general, to a worsening of anxiety, depression, and insomnia, and that the most affected gender was female, while the age group most at risk was that between 50 and 65 years old [112]. 

Reduction in or lack of social support has been demonstrated to be a determining factor in increasing anxiety levels in older people and those with cognitive impairment, as well as in reducing well-being among the elderly and carers [113]. 

Older adults, however, also presented protective factors, such as a more concrete vision of events, less emotional reactivity, and a favorable disposition concerning the future [114].

## 4. Discussion

The purpose of the present review was the collection of the studies that have investigated, in the European context, the impact of the SARS-CoV-2 prevention measures on mental health in the general population. The following step was constituted by analyzing results for different age groups, published from 2021. The choice of the study period was guided by the need to obtain reliable data both on the various pandemic waves (epidemiology and restrictions) and on the evolution of clinical manifestations. The evidence for 2020 was derived from studies published starting from 2021, which, presenting more extended study periods, offered a general vision and more complete data in terms of symptomatic evolution, psychiatric manifestations, and relationship with the different phases of the global emergency.

In the children group, an increase in the prevalence of psychopathological symptoms, such as behavioral changes, sleep disorders, anxiety, and related symptoms, has been observed. Behavioral changes also tended to increase progressively over time, unlike emotional changes, which remained stable.

Among adolescents, about one-third of those surveyed during the pandemic experienced a prevalence of anxiety and depression symptoms. These results were similar to findings by Śniadach et al. [115] on an increase from one to four times of depressive and anxiety symptomatology. 

In addition to the described symptoms, both children and adolescents have presented several behavioral changes that have impaired their well-being: alterations in the daily routines and the sleep–wake cycle, problematic use of the internet, a reduction in healthy sports habits, and a change in lifestyle and sociality.

Among minors, the presence of past neurodevelopmental disorders and psychopathological problems or emotional dysregulation represent an important risk factor for the increase in psychological problems.

Further predisposing conditions have been the presence of intra-family relational problems, socio-economic difficulties, and poor social support.

Among both adolescents and adults, the female gender was more at risk of post-traumatic suffering, anxiety, depression, and increased suicidality.

These data reflect those collected from extra-European studies [116] showing that women have more psychological suffering than their male counterparts, with a higher probability of post-traumatic stress [117], anxiety, and depression [118].

Regarding the female gender, some studies have evidenced that neuroticism and basal levels of anxiety and depression were responsible for psychological alteration during the pandemic [83]. These data confirm what is already known in the literature about the higher prevalence rates among women of anxiety disorders [119] and depression.

The factors usually considered in the explanation of such gender differences are the “sex role”, that is education-determined tendency to internalization, rather than to resilience, social factors—with the role of a woman discriminated against or subjected to greater risk of victimization than that of a man—and biological factors [120]. 

However, data on this gender gap during the pandemic period highlighted the importance of social factors and the condition of loneliness, as well as a greater burden of family responsibilities and duties [121].

Younger age, in general, has been found to constitute an undoubted factor of risk for a greater psychological burden during the lockdown period. This concerned both the youngest among adolescents and children and the youngest among adults and the elderly, as confirmed by longitudinal studies.

Several factors have been suggested to explain this difference. Teens, via social media, can have easier access to news about critical events, resulting in more stress. On the other hand, it was also found that the increased specific knowledge about the pandemic and preventive measures protected against psychological distress.

In general, children and adolescents likely have much more uncertainty about their future and less emotional and cognitive protective abilities to cope with stress [122,123]. In addition, they suffer greatly from social isolation from peers and school, with the possibility of an increase in intra-family conflict [124]. 

In such groups, social connectedness represents a positive moderator of depressive symptoms, anxiety, and life satisfaction. Additionally, this factor should be supported by adults to avoid the risk of internet or smartphone addiction.

It should be considered that adolescents are naturally looking for new experiences, and this can expose them to the risk of developing addictions, as well as substance use [125].

However, when supported, youth have proactivity to learn and communicate, including through technological platforms such as that used in the healthcare sector.

As regards young adults, a great concern is represented by work and financial problems, exacerbated by pandemic limitations [126].

Unemployment, difficulties or changes in work, a low socioeconomic level, and an unfavorable milieu are the main risk factors of psychological distress for adults.

The results referring to the European context mirror those found overall at the extra-European level, suggesting that similar stressors represent a homogenous risk factor regardless of the socio-economic specificities of individual countries [127].

Finally, as regards individual psychological aspects, loneliness is among the main risk factors for adolescents, adults, and the elderly for both the development and the maintenance of poor mental health outcomes.

Difficulties in family relationships also represent predictors of anxiety and depression symptoms for all age groups. 

Some personality traits, such as neuroticism, the use of maladaptive coping strategies, emotional suppression, and especially the presence of a mental state at risk or a history of mental illness, may affect psychological well-being during the pandemic.

In perspective, although some longitudinal studies have lowered the prediction of the risk of a pandemic resulting in a psychological “tsunami” following the viral pandemic phase and related restrictions, it must be said that not all studies agree. In this sense, many mental health professionals have expressed strong concern about what may happen, especially to children and adolescents.

Data collected by Revet et al. [128] showed that, according to the European Society for Child and Adolescent Psychiatry (ESCAP), the perceptions of practitioners about the mental health of children and adolescents had significantly worsened compared to the period before the pandemic.

While in April/May 2020 more than half of the respondents perceived the impact of the crisis as “medium”, in February/March 2021, more than 80% of the respondents perceived the impact of the crisis as “strong” or “extreme”.

Although restrictive measures have ceased in most parts of the world, this does not determine that, in terms of mental health, the impact of the pandemic crisis is over. Indeed, some studies have shown the persistence of symptoms even in the period strictly following the end of the lockdown and, consistently severe, in people with a mental disorder already before the pandemic.

In addition, it has been found that the repetition of the waves of infection and the related restrictive measures is accompanied by an exponential increase in mental disorders. This event is a sign of increased psychopathological vulnerability and a reduction in emotional and material resources for coping with stress. Since the possibility of new waves is not excluded in the future, this should certainly be considered.

Women and young people, especially in cases of high degrees of loneliness and poor spiritual well-being, were found to be more at risk of a poor psychological outcome at the end of the lockdown.

Loneliness is, especially for older people, a determining factor in the risk of developing psychic distress.

Moreover, it should be considered that social isolation is a predictor of mortality, as are other well-documented clinical variables, such as hypertension, obesity, hypercholesterolemia, and smoking [129]. 

The reduction in social support represented a key factor in increasing anxiety levels in older people and those with cognitive impairment, as well as reducing well-being in the elderly and caregivers. This underlines the importance of social support, which has proved to be an important protective factor, together with the degree of resilience, towards the development of suicidal ideation.

Regarding suicide, an increase in attempts by 25% was observed among adolescents, with a higher risk in the case of the female gender. This risk also increased among adults, particularly in the presence of symptoms of anxiety and depression, together with unfavorable socio-environmental conditions. These results mirror findings in non-European studies [130]. 

It has been suggested that social isolation is an even greater psychological burden for adolescents than for adults.

Social support has, therefore, proved to be an important element for all age groups, albeit in a diversified way. For example, in the adult group, job support was a priority. It has been observed that, where economic policies pursued assistance for unemployed or dismissed workers, there has been no significant deterioration in mental health [131].

Finally, the review of the most recent European studies has highlighted findings that agree with those worldwide.

## 5. Implications

The present review showed that some population groups have been at greater risk of mental suffering during pandemic waves and certainly represent priority targets for social and health intervention. However, it should be noted that, in general, the identification of social groups as “vulnerable” may also represent an element of discrimination. The word “vulnerable” could be misunderstood and elicit stigmatization of people as “different” and “at-risk”. This would contribute to the isolation of stigmatized groups and the worsening of the psychological burden. In the case of the SARS-CoV-2 pandemic, it sometimes seemed that a “generational war” was being conducted, where the restrictive measures strongly suffered by the youngest, who were less at risk in terms of physical morbidity and mortality, were adopted to protect the physically vulnerable elderly population. Thus, it would be useful to stimulate closeness and the exchange of support between different generations. Older people, for example, could transmit to youth an optimistic view of the future and their concrete abilities in coping with a stressful situation, such as the pandemic.

In general, it might be useful to provide citizens an overall view of the pandemic problems rather than classifying them into less or more mentally or physically vulnerable groups [132]. The media could certainly play an important role in this respect, showing how the risk of pandemic spread is a danger for all age groups [133,134].

The pandemic has represented, especially in the initial stages, an unexpected event to which it was necessary to respond in a collective manner, resulting in a reinforcement of collective resilience. It must be said, however, that, over time, many pressures have developed towards the disintegration and discrimination of social groups. Initially, there was ethnic prejudice, originating from Asian regions; later, there was antagonism between age groups. Finally, there was a clash between supporters and opponents of mass vaccination, in addition to the stigma of infected people [135,136].

Strategies to counteract these phenomena should, therefore, be prepared as a global approach for all citizens. Naturally, evidence from the studies elicits the possibility of multiple and diversified interventions to avoid exclusions and discrimination.

In any case, it should be considered that trying to improve general population levels of physical and mental health, social inclusion, as well as economic and work support, represents an active way to prevent critical events, such as the consequences of infectious spread.

**Figure 2:**
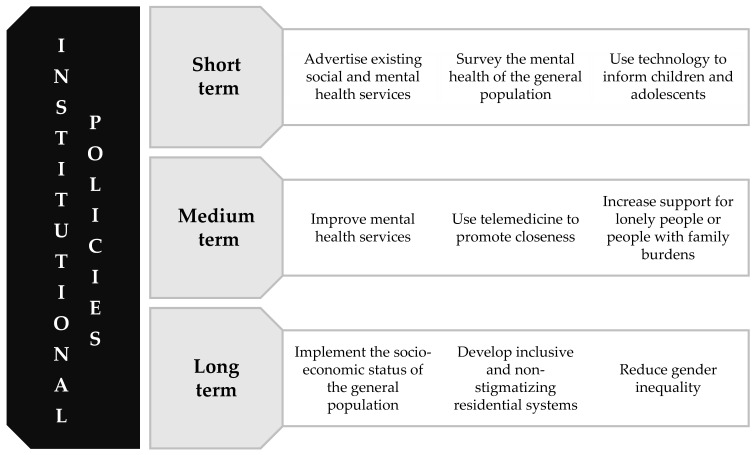
Suggestions for socio-health policies to support the general population.

The pandemic period has confirmed that the protection of mental health, both for people with mental disorders and for the general population, cannot be separated from institutional attention to a comprehensive set of interventions (Figure 2) [137,138]. In fact, mental health is the product of multiple social, economic, biological, psychological, and cultural interactions.

Furthermore, technological resources have been demonstrated to be a useful tool both in school, work, and in the health context.

## 6. Conclusions

The research carried out provided an updated summary of the main studies conducted on the mental health status of the general population during the pandemic, highlighting the most expressed categories of disorder, risk factors, evolution, and, subsequently, the groups needing specific interventions, both in the ongoing pandemic phase and in the event of possible future restrictions. Following the analysis of the data available in the international literature, it was also possible to identify a series of recommendable institutional interventions that could be developed. Moreover, territorial support and the use of new technologies should be considered due to logistical advantages, such as capillarity and quickness, as tools able to constitute key factors in the field of mental health care.

Regarding the limitations of the review, most of the included studies had a cross-sectional design. Only a few longitudinal studies have made a comparison with the periods before the pandemic or analyzed the differences between the waves and the related social restrictions. Nonetheless, a significant proportion of the studies were based on online surveys, which represent a method not uniquely considered to be realistically descriptive of the situation under investigation [139,140].

Furthermore, the different studies were heterogeneous in samples, objectives, and methods. Despite these limitations, the analyzed studies converge towards similar evidence, substantially in agreement with the extra-European international results.

## Data Availability

The data that support the findings of the study are available from the corresponding author upon reasonable request.

## Supplementary Materials

The following supporting information can be downloaded at: https://www.mdpi.com/article/10.3390/ijerph19159347/s1, Table S1: Children and Adolescents; Table S2: Adults; Table S3: Elderly.
