# Supplementary material for: Mental Health Consequences of COVID-19 Pandemic Period in the European Population: An Institutional Challenge
Source: Int J Environ Res Public Health. 2022 Jul 30;19(15):9347. doi: 10.3390/ijerph19159347 (PMC9367746; doi:10.3390/ijerph19159347)
Supplement: Supplementary file 1 [file ijerph-19-09347-s001.zip › ijerph-1815291-supplementary.pdf]

Table S1. Children and Adolescents.

Author<br>Year	Country<br>Design<br>N, Age	Main Mental Health Measures (totally or in part)	Main findings
Meda et al., 2020 [61]	- Italy<br>- Longitudinal<br>- N. 358, 18–30 y	- Beck Depression Inventory – 2 (BDI-2)<br>- Beck Anxiety Inventory (BAI),<br>- Obsessive-Compulsive Inventory-Revised (OCI-R)<br>- Eating Habits Questionnaire (EHQ)<br>- Eating Disorder Inventory-3 (EDI-3)	- Patients reported on average worse depressive symptoms during lockdown than 6 months before isolation.<br>- About 6% could develop more severe depressive symptoms.
Mohler-Kuo et al., 2021 [45]	- Switzerland<br>- Cross-sectional<br>- N. 1146; 12–17 y<br>- N. 1627; 19–24 y	- Generalized Anxiety Disorder 7 (GAD-7)<br>- Patient Health Questionnaire-9 (PHQ-9)<br>- Patient Health Questionnaire-9 (PHQ-9)<br>- Kiddie Schedule for Affective Disorders and Schizophrenia (K-SADS)<br>- Spence Children's Anxiety Scale for Children (SCAS-C)<br>- Responses to Stress Questionnaire (RSQ)–(COVID-19)	- About 1/3 of patients screened positive for at least one of the mental health problems (attention deficit hyperactivity disorder, oppositional defiant disorder, depression, anxiety) and problematic internet use.
Hu and Qian, 2021 [55]	- UK<br>- Longitudinal<br>- N. 886; 10-16 y	- Strengths and Difficulties Questionnaire (SDQ)	- Distinct effects of lockdown on mental health problems: amelioration in adolescents with poor mental health before and worsening in adolescents with

			relatively good mental health before.<br>- Risk factors for poor mental health: one-parent, one-child, and low-income families.
Gracia et al., 2021 [54]	- Spain<br>- Longitudinal<br>- N. 690; 12–18 y	- Data from the Catalonia Suicide Risk Code (CRSC),	- Adolescents with Suicide Attempts (SA) represented a 25% increase compared to the previous year (n. 552)<br>- Gender difference: SA rates were constant in boys (32.1–32.3/100.000), and substantially increased in girls (from 99.2 to 146.8/100.000).
12. Cellini et al., 2021 [42]	- Italy<br>- Cohort<br>- N. 299 mothers reported on their children (6–10 y)	- Pittsburgh Sleep Quality Index (PSQI)<br>- Sleep Disturbance Scale for Children (SDSC)<br>- Subjective Time Questionnaire<br>- Strengths and Difficulties Questionnaire–Parent version (SDQ-P)<br>- The Strengths and Difficulties Questionnaire—18+ (SDQ 18+)<br>- The Difficulties in Emotion Regulation (DERS)	- Mild worsening in children's sleep quality and increased emotional, conduct, and hyperactive symptoms.<br>- Difficulties in children were predicted by their sleep quality (i.e., worse sleep quality, worse emotional difficulties), their increasing boredom, and the mothers' emotional symptoms and difficulties.
Burdzovic Andreas and Brunborg, 2021 [51]	- Norway<br>- Cohort<br>- N. 1,621 before the pandemic; N. 915 during the pandemic; 13-19 y	- Data from MyLife study (annual electronic surveys)<br>- Patient Health Questionnaire-9 (PHQ-9)	- Lower odds of organized sports participation in the COVID-19 cohort.<br>- Adolescents with high pandemic anxiety were more likely to experience clinical-level depression symptoms and poor physical health.

Mastorci et al., 2021 [52]	- Italy<br>- Longitudinal<br>- N. 1,019; 10-14 y	- Personalized Well-Being Index (PWBI)<br>- KIDSCREEN-52<br>- Mediterranean Diet Quality Index for children and adolescents (KIDMED)<br>- Physical Activity Questionnaire for Older Children (PAQ-C)	- General decrease in well-being perception, expressed in lifestyle habits, social, and emotional components.<br>- Some improvement in the cognitive skills
Raw et al., 2021 [48]	- UK<br>- Longitudinal<br>- N. 2,988 4-16 y	- Strengths and Difficulties Questionnaire (SDQ)<br>- Depression Anxiety Stress Scales (DASS-21)	- Levels of hyperactivity and conduct problems increased over time, whereas emotional symptoms remained stable.<br>- Risk factors for poor outcome: parents/carers with higher levels of psychological distress, history of Special Educational Needs (SEN)/Neurodevelopmental Disorders (ND), younger age.
Ertanir et al., 2021 [59]	- Switzerland<br>- Longitudinal<br>- N. 377, 11-13 y	- Hopkins Symptoms Checklist (HSCL-25)<br>- Adolescent Stress Questionnaire (ASQ-S)	- Detrimental effects noticeable only during containment measures.<br>- Female gender predicted more depression and anxiety levels.
Myhr et al., 2021 [53]	- Norway<br>- Cohort<br>- N. 2,443, 13-16 y	– Hopkins Symptom Checklist (HSCL-25)<br>– Questionnaire measuring Loneliness, Life Satisfaction, Quality of Life, Socioeconomic Position	– Decrease in high quality of life and life satisfaction.<br>– Female gender was associated with higher odds of reporting high depressive symptoms.<br>– Rising rates of psychological distress among the least privileged socioeconomic groups.
Rauschenberg et al., 2021 [49]	- Germany<br>- Cohort<br>- N. 666, 16-25 y	– Three-Item Loneliness Scale<br>– Kessler Psychological Distress Scale (K10)	– 38% of youth met the criteria for moderate or severe psychological distress.

		– Questionnaire measuring COVID-19-related cognitive preoccupation, worries, and anxiety; sociodemographic status; current use of, and attitudes toward mobile health (mHealth) apps	– Psychological distress was progressively more likely to occur as levels of social isolation increased.<br>– Psychological distress, worries, and anxiety were associated with a positive attitude toward using mHealth interventions.
Monnier et al., 2021 [41]	– France<br>– Cross-sectional<br>– N. 5,702, 8-9 y	– Strengths and Difficulties Questionnaire (SDQ),<br>– Questionnaire on children's health behavior, schooling, socioeconomic status of families	– Children's sleeping difficulties were associated with children's abnormal symptoms of both hyperactivity-inattention and emotional symptoms.<br>– Factors specifically associated with abnormal hyperactivity/inattention: male sex, access to specialized care prior to the pandemic and its suspension during school closure, abnormal emotional symptoms, being unschooled or schooled with assistance before lockdown, and tutoring with difficulties or absence of a tutor.<br>– Factors associated with children's emotional symptoms were the following: being born pre-term, COVID-19 cases among household members, abnormal symptoms of hyperactivity/inattention, and a modest income.
Pisano et al., 2021 [50]	– Italy<br>– Cross- sectional<br>– N. 326, 14-19 y	– State-Trait Anxiety Inventory (STAI)	– Prevalence of 47.5 % of anxiety and 14.1% of depressive symptoms.

		– Mood and Feelings Questionnaire-short form (MFQ-SF)<br>– Strength and Difficulties Questionnaire (SDQ)	– From regression analyses resulted: previous psychopathological status and worries about infection were linked to anxiety; female gender, previous psychopathological status (moderated by the change in lifestyle); the worse environmental context was linked to depression.
Matalí-Costa and Camprodon-Rosanas, 2022 [47]	– Spain<br>– Cross- sectional<br>– N.850, 4-18 y	– Pediatric Symptom Checklist, Massachusetts General Hospital scale (PSC)	– Living in a home smaller than 80 m^2 was associated with a risk of emotional or behavioral distress<br>– Age inversely correlated to behavioral and emotional functioning, as the youngest group of children (4–8 years) presented more anxiety. fear, and behavioral expressions of this in the form of irritability or restlessness
Luijten et al., 2021 [43]	– Netherlands<br>– Cohort<br>– N. 2,401 (2018) 8-18 y<br>– N. 844 (2020) 8-18 y	– Six Dutch-Flemish Patient-Reported Outcome Measurement Information System (PROMIS) pediatric measures:<br>– V2.0—Anger,<br>– CAT V2.0—Peer Relationships,<br>– V1.0—Global health (7 + 2),<br>– CAT V1.0—Sleep-related Impairment,<br>– CAT V2.0—Anxiety<br>– CAT V2.0—Depressive Symptoms	– During the lockdown, more children reported severe Anxiety and Sleep-Related Impairment.<br>– Associated factors with worse mental/social health were single-parent family, having three or more children, a negative change in the work situation of parents due to COVID-19 regulations, and a relative/friend infected with COVID-19.

Pizarro-Ruiz and Ordóñez-Camblor, 2021 [46]	– Spain<br>– Cross- sectional<br>– N.325, 8-12 y<br>– N. 265, 12-18 y	– Scales of Assessment System for Children and Adolescents (SENA)	– Strict confinement situation of children and teenagers already revealed, from 8 to 10 days, significant consequences on the mental health.<br>– Children experienced more rebellious behaviours and a worsening in rage control and emotional regulation.
Thorisdottir et al., 2021 [58]	– Iceland<br>– Longitudinal<br>– N. 59,701, 13-18 y	– Symptom Checklist-90<br>– Short Warwick Edinburgh Mental Wellbeing Scale<br>– Questionnaire on cigarette smoking, e-cigarette use and alcohol drinking	– Depressive symptoms increased and mental well-being worsened in all age groups.<br>– Cigarette smoking, e-cigarette use and alcohol intoxication declined.
Essau and de la Torre-Luque, 2021 [57]	– UK<br>– Cohort<br>– N. 904, mean age = 19.17 y at T0 (2018-2019) and 904 participants mean age = 19.17 y at T1 (2020)	– Data from the MCS sweep 7 survey and the COVID-19<br>– Survey administered to MCS cohort members<br>– Strength and Difficulties Questionnaire (SDQ)<br>– 3-item version of the Social Provisions Scale<br>– 3-item UCLA Loneliness Scale (UCLA T-ILS)<br>– K6 Kessler Distress Scale<br>– Warwick-Edinburgh Mental Wellbeing Scale (WEMWBS)<br>– Patient Health Questionnaire (PHQ-2)<br>– Generalised Anxiety Disorder Scale (GAD-2)	– Adolescents in the ”high-symptom” and “emotion-dysregulation” classes had the worst outcome during the lockdown: more stress, conflict and loneliness, and lower levels of perceived social support; adolescents in the emotional-dysregulation class also consumed more alcohol and had a worse financial situation during the lockdown compared to pre-lockdown period.

Revet et al., 2021 [128]	– Europe (22 countries)<br>– Longitudinal<br>– N. 60 heads of CAP (European Society for Child and Adolescent Psychiatry) university services	– Self-report online questionnaire survey	– Respondents' major concerns related to CAP: difficulties in managing the increased numbers of patients (83%), difficulties in managing families in situations of high psychosocial precariousness (83%), and reduced financial resources for CAP services (48%).<br>– The perceived impact on the mental health and psychopathology of children and adolescents dramatically increased from "medium" (>50%) in 2020 to "strong" or "extreme" (80%) in 2021.<br>– Four nosographic entities were particularly impacted: suicidal crises, anxiety disorders, eating disorders, and major depressive episodes.
Śniadach, J., 2021 [115]	– Poland, Germany,Italy, Spain, China, USA<br>– Review	– Literature Review	– 1 to 4-fold increase in prevalence of depression and anxiety disorders among children and adolescents internationally.
Garcia-Adasme et al., 2021 [44]	– Spain<br>– Cross-sectional<br>– N. 2,292, 0-17 y<br>–	– Children's Manifest Anxiety Scale (CMAS) for children older than 7 years<br>– A questionnaire on children's behavioural, emotional/somatic symptoms and family environment, reported by	– 56.3% of the children below 7 years had four or more anxiety-related symptoms, the most frequent of which were tantrums, emotional changes, restlessness, and fear of being alone.<br>– Children between 7 and 17 years, boys, in particular, scored high on

		parents, for children younger than 7 years	the anxiety spectrum: 43,8% resulted above the 75th percentile.<br>– Significantly high values were found in all aspects of anxiety among those who feared infection or whose parents were unemployed.<br>– Having someone in the family home infected with COVID-19 was a trans-age risk factor.

Table S2. Adults.

Author<br>Year	Country<br>Design<br>N, Age	Main Mental Health Measures (totally or in part)	Main findings
Bonati et al., 2021 [70]	– Italy<br>– Cross-sectional<br>– N. 20,158, 18-50 y	– COVID-19 Peritraumatic Distress Index (CPDI)	– Increasing CPDI score was associated with gender (female), first-second educational level, being unemployed, living in a ≤2 room house, having had new health problems during the previous 14 days, and not having been out of the house in the previous week.
Liu et al., 2021 [71]	– Germany<br>– Longitudinal<br>– N. 1,903, 18-81 y	– COVID-19 Peritraumatic Distress Index (CPDI)<br>– UCLA Loneliness Scale short-form (ULS-8)	– Prevalence of psychological distress caused by the COVID-19 pandemic significantly rose from 24% to 66% between the peak and off-peak transmission period.<br>– Unemployment rate and loneliness increased negative mental health outcomes.<br>– Psychological distress scores increased mostly in female, young, and lonely people.
Mata et al., 2021 [62]	– Germany<br>– Longitudinal<br>– N. 3,500, 16-75 y	– State-Trait Anxiety Inventory short scale<br>– Patient Health Questionnaire (PHQ-2<br>– Questionnaire on screen time, eating habits, physical activity,	– Symptoms of anxiety, depression, and loneliness were highest shortly after the lockdown came into effect.<br>– Over time, the symptoms were stable or went down slightly.<br>– More screen time, more snacking, and less physical activity were

			related to higher symptoms of anxiety, depression, and loneliness across all time points.<br>– The proportion of women meeting physical activity recommendations stayed below pre-lockdown levels, even three months after the lockdown started.
Fiorenzato et al., 2021 [63]	– Italy<br>– Cross-sectional<br>– N. 1,215, 18-88 y	– Prospective and Retrospective Memory Questionnaire (PRMQ)<br>– 10-item questionnaire created to assess the subjective global cognitive functioning in performing everyday tasks<br>– Hospital Anxiety and Depression Scale (HADS)<br>– Items 16, 18 and 21 of the Beck Depression Inventory (BDI-II) on appetite, sleep and interest in sex	– Detrimental effect on attention, temporal orientation, and executive functions, especially in the female gender, younger age, and home confinement.<br>– Higher prevalence (32.30% vs 15.39% pre-lockdown) and severity (moderate/severe cases 13.71% vs 4.35% pre-lockdown) of depression.<br>– Higher prevalence (35.72% vs 21.40% pre-lockdown) and severity (moderate/severe cases 16.86% vs 6.69% pre-lockdown) of anxiety disorders.<br>– Abnormal sleep, appetite changes, reduced libido and health anxiety.<br>– Risk factors for worsening cognition and mental health: being female, under 45 years, working from home or underemployment
Lorant et al., 2021 [72]	– Belgium<br>– Cohort<br>– N. 20,792, 15- 75+ y	– General Health Questionnaire-12 (GHQ-12)	– 52.9% of respondents experienced psychological distress after less

		– Social Participation Measure (SPM)<br>– Oslo Social Support Scale (OSS-3)<br>– Short Loneliness Scale (LON)	than a week of confinement (on average 5.6 days).<br>– Risk factors: longer period of confinement, female sex, younger age, exposition to COVID-19, changes in occupational status, decrease in social activity and support.
Gröndal et al., 2021 [89]	– Sweden<br>– Cross-sectional<br>– N.471, 18-75 y	– Brief Irritability Test (BITe)<br>– Emotion Regulation Questionnaire (ERQ)<br>– State-Trait Anger Expression Inventory-2 (STAXI-2)<br>– Satisfaction With Life Scale (SWLS)<br>– UPPS Impulsive Behavior Scale	– Unemployed respondents reported having suffered more severe consequences for their work/study life and finances.<br>– Respondents with a higher tendency to experience and express anger, and those with a higher level of impulsivity were more likely to report a negative impact on their family life, their work or study life, and their finances.<br>– The relationship between the impact on social life, free time activities, physical activity, and subjective well-being appeared to be moderated by respondents' disposition for cognitive reappraisal.
Andersen et al., 2021 [76]	– Denmark<br>– Longitudinal<br>– N. 1,526, 18-79 y	– World Health Organization Five Well-being Index (WHO-5)<br>– Work and Social Adjustment Scale (WSAS)	– Reduction of depressive symptoms and functional impairment immediately after the lockdown specifically among adults with children living at home.

			– Longitudinal protective effect on functional impairment by living with children.
Pérez et al., 2020 [64]	– Spain<br>– Cross-sectional<br>– N. 1,781, 18-91 y	– Brief Symptom Inventory 18 (BSI-18)<br>– Emotional Regulation Questionnaire (ERQ)<br>– Emotional Regulation Questionnaire (ERQ)<br>– Insomnia Severity Index (ISI)<br>– Visual Analogue Scale (VAS)	– 25%-39% of patients referred clinically significant levels of distress.<br>– 29.1% of patients showed clinically significant levels of depression, 15.8% anxiety, and 23.8% somatization.<br>– Women showed higher levels of distress, negative affect, perception of pain, and cognitive reappraisal, lower levels of emotional suppression, and sleep quality.<br>– Loneliness, living in flats, and younger age were related to higher distress.<br>– Cognitive reappraisal was related to lower distress.
Papadopoulou et al., 2021 [69]	– Greece<br>– Cross-sectional<br>– N. 5,116, 18-65+ y	– Generalized Anxiety Disorder scale (GAD-2)<br>– Patient Health Questionnaire (PHQ-2)<br>– Systemic Clinical Outcome and Routine Evaluation (SCORE-15)<br>– Connor-Davidson Resilience Scale (CD-RISK-2)	– 5.20% of patients reported suicidal thoughts, 14.17% anxiety, and 26.51% depression.<br>– Unmarried or divorced marital status, mental health history, poor perceived quality of physical health, impaired family functioning, anxiety, and depression symptoms were independently associated with higher odds of suicidal ideation.<br>– Higher resilience, positive feelings towards lockdown measures, relationship with friends, and

			spirituality were associated with lower suicidal ideation odds
Muro et al., 2021 [83]	– Spain<br>– Longitudinal<br>– N.155, 16-76 y	– Beck Depression Inventory-II (BDI-II)<br>– State-Trait Anxiety Inventory (STAI)<br>– Big Five Inventory-10 (BFI-10)<br>– Questionnaire on health-behavior including physical activity	– Lockdown duration, increased neuroticism, and baseline levels of anxiety and depression were risk factors for women's mental health.<br>– Routines and physical activity emerged as protective factors for managing psychological wellbeing during the pandemic lockdowns.
Marmet et al., 2021 [90]	– Switzerland<br>– Longitudinal<br>– N. 2,345, mean age 29 y (SD = 1.28)	– Data from Cohort Study on Substance-Use Risk Factors (C-SURF)<br>– Major Depression Inventory (WHO–MDI)<br>– Perceived Stress Scale (PSS)<br>– Pittsburgh Sleep Quality Index(PSQI)<br>– Impact of Event Scale (IES)<br>– Questionnaire on feelings about COVID-19	– A relatively low socioeconomic status already before the crisis was a risk factor for a higher psychological impact in terms of major depression, perceived stress, psychological trauma, and isolation.<br>– Other risk factors: unemployment or partially employment, increased workload, or work from home.
Ribeiro et al., 2021 [84]	– Luxembourg<br>– Longitudinal<br>– N. 1,756, 18-84 y	– Center for epidemiologic studies depression scale (CES-D)<br>– Generalised anxiety disorder 7-item (GAD-7)<br>– UCLA Loneliness Scale short-form (ULS-8)<br>– Perceived stress scale – 4 item version (PSS-4)	– Risk factors for the decrease in mental health (stress, depression, anxiety): female sex, younger age, poor socio-economic status.
Burrai et al., 2021 [85]	– Italy<br>– Longitudinal<br>– N. 492, 18-24	– Questionnaire on demographic characteristics, knowledge, perceptions, and behaviors related to COVID-19	– High prevalence of experiencing unprecedented disruptions in daily lives, leading to isolation and loneliness.

		– Depression, Anxiety, and Stress Scale–21 items (DASS-21)<br>– COVID-19 Prevention, Recognition, and Home-Management Self-Efficacy Scale<br>– Self-reported preventive behavior and motivation to engage in preventive behavior scale<br>– Social Connectedness Scale (SCS)	
Bendau et al., 2021 [96]	– Germany<br>– Longitudinal<br>– N. 6,551, mean age = 36.94 y (SD = 11.70)	– COVID-19-Anxiety Questionnaire (C-19-A)<br>– Patient Health Questionnaire-4 (PHQ-4)<br>– Questionnaire on mental health status	– COVID-19-related fear, depressive, and anxiety symptoms were on average higher in individuals with (vs. without) depression, anxiety, or other mental disorders.<br>– All symptoms decreased in the total sample over time, strongly in individuals with anxiety vs. depressive disorders.
Vrublevska et al., 2021 [73]	– Latvia<br>– Cross-sectional<br>– 2,608; mean age of men = 48.04 (SD=13.63); mean age of women = 44.74 y (SD= 15.32)	– Center for Epidemiologic Studies Depression (CES-D)<br>– Questionnaire on mental health status and spirituality<br>– Risk Assessment of Suicidality Scale (RASS)	– Prevalence of distress was 7.82%; prevalence of depression among women was 7,.22% and 4.21% among men.<br>– Participants with depression were significantly younger than those who were not depressed.<br>– Non-depressed men were significantly older than non-depressed women.<br>– Living in the capital (Riga) or in other urban areas was a risk factor for more distress than living in rural areas (9.32% and 7.85% vs. 6.26% for distress and 5.36% and 7.40% vs 4.37% for depression).

			– Suicidal thoughts increased in 13.30% of those with a history of clinical depression, and in 27.05% of those with a history of suicidal attempts.<br>– Unemployed people were more likely to have clinical depression or distress (13.68% and 10.38%) compared to those who were employed (7.38% and 5.27%) or economically inactive (7.06% and 5.55%).<br>– Twenty-one significant variables were found to contribute to the changes in anxiety, depressive thoughts, thoughts of suicide, and current distress or depression.<br>– Protective factors included positive changes in family relationships and economic situation, maintaining one's basic routine, and having more people living in the household.
Hubbard et al., 2021 [74]	– Scotland<br>– Cross-sectional<br>– N. 1,006, 34-65 y	– Telephone interview on sensitive topics including mental and general health.<br>– Patient Health Questionnaire (PHQ-4)<br>– Scottish Index of Multiple Deprivation (SIMD)<br>– ENRICHD Social Support Instrument (ESSI)	– Mental health was poorer in younger adults, women, and people living in the most deprived areas.<br>– Age effects were exacerbated by loneliness and illness (Covid-19) representations; gender effects by loneliness and illness (Covid-19) representations; deprivation effects by loneliness, social support, illness (Covid-19)

			representations, and perceived threat.
Groarke et al., 2021 [93]	– UK<br>– Longitudinal<br>– N. 1,958, 18-87 y	– UCLA Three-Item Loneliness Scale (UCLA T-ILS)<br>– Patient Health Questionnaire (PHQ-4)<br>– Difficulties in Emotion Regulation Scale - Short Form (DERS-SF)	– Loneliness predicted higher depressive symptoms one month later, and depressive symptoms predicted higher loneliness one month later.<br>– Emotion regulation difficulties and depressive symptoms were reciprocally related over time.
Kalaitzaki et al., 2022 [65]	– Greece<br>– Longitudinal<br>– N. 1,009, mean age 36.6 y (SD = 12.9) T1 and 32.0 y (SD = 12.9) for T2	– Questionnaire on participants' experiences during the lockdown<br>– PTSD Checklist for DSM-5 (PCL-5)<br>– Perceived Stress Scale 10-item version (PSS-10)<br>– Brief Resilience Scale (BRS)<br>– Brief Coping Orientation to Problems Experienced Inventory (COPE)<br>– Revised UCLA Loneliness Scale (R-ULS)<br>– ENRICHD Social Support Instrument (ESSI)	– Clinically significant levels of post-traumatic stress symptoms (PTSS) were presented by 26.1% and 35.5% of the participants during T1 and T2 respectively.<br>– Higher levels of loneliness, use of maladaptive coping strategies, lower levels of social support and resilience, and use of adaptive coping strategies were frequent.<br>– During both lockdowns, PTSS were predicted by perceived stress, loneliness, reduced resilience, and the coping strategies of denial and self-blame.
Reme et al., 2022 [60]	– Norway<br>– Longitudinal<br>– N. 88,041, mean age 46.4 y (SD = 5.3).	– Data from the Norwegian Mother, Father and Child Cohort Study (MoBa)<br>– Hopkins Symptom Checklist-5 (SCL-5)	– High increases in depressive symptoms with little evidence of unequal distribution across socioeconomic status. Such an increase was particularly strong among women and those with lower levels of depressive symptoms prior to COVID-19.

Benatov et al., 2022 [99]	– Germany, Israel, Poland, and Slovenia<br>– Longitudinal<br>– N. 1,724, 20-40 y	– PTSD Checklist-Specific Version (PCL-S)<br>– Perceived Stress Scale (PSS-10)<br>– Generalized Anxiety Disorder (GAD-7)<br>– Extended version of Patient Health Questionnaire (PHQ-9), with an item introduced to evaluate suicidal/self-harm ideation (PHQ-9)<br>– Questionnaire on physical activity	– Single individuals, students, and parents in young adulthood across all countries were mostly at risk for health problems.<br>– A younger age (20–29 years) predicted coronavirus-related PTSD risk, whereas the female gender predicted high stress.
Medda et al., 2021 [75]	– Italy<br>– Longitudinal<br>– N. 1,690, 18-93 y	– Data from the Italian Twin Registry (ITR) survey<br>– three-item Oslo Social Support Scale (OSS-3)<br>– two-item Patient Health Questionnaire (PHQ-2)<br>– 9-item version of the Patient Health Questionnaire (PHQ-9)	– Prevalence of depressive symptoms increased from 33.6 to 38.9% immediately after lockdown.<br>– Younger, less educated people, and women were at greater risk of developing depressive symptoms.
Pashazadeh Kan et al., 2021 [92]	– Systematic review	– Studies focusing on anxiety prevalence among the general population	– Europe revealed the highest prevalence of anxiety at 54.6% (95% CI, 42.5%; 66.2%).
Andersen et al., 2021 [76]	– France<br>– Longitudinal<br>– N. 662, mean age 39.8 y (SD = 3.65)	– Data from TEMPO (Trajectoires Épidémiologiques en POpulation) cohort with information on sociodemographic data and preexisting symptoms of anxiety/depression<br>– Mini-International Neuropsychiatric Interview (MINI)	– Female sex, low household income, and loneliness were risk factors for anxiety/depression during the lockdown.

		– Anxious/Depressed syndrome scale based on the Adult Self Report (ASR)-Achenbach System	
Kekäläinen et al., 2021 [77]	– Finland<br>– Longitudinal<br>– N. 358 (only women), 51-59 y	– Data from Estrogenic Regulation of Muscle Apoptosis (ERMA) study<br>– Centre for Epidemiological Studies Depression Scale (CES-D)<br>– Short form of the Eysenck Personality Inventory (EPQR-S)<br>– Questionnaires about depressive symptoms, eating behavior, physical activity, and alcohol consumption	– Women reported more depressive symptoms and unhealthier eating habits at the end of the emergency conditions compared to the pre-pandemic time.<br>– Higher extraversion was associated with a perceived decrease in alcohol consumption and with changing to healthier eating habits.
Cárdaba-García et al., 2021 [66]	– Spain<br>– Cross-sectional<br>– N. 808, 18-80 y	– Goldberg Anxiety and Depression Scale (GADS)	– 63% of the participants were at risk of suffering from anxiety and 64.9% were at risk of depression.<br>– Risk factors for anxiety and depression: younger age, female sex, single status, previous need for psychological help and need for such help at the time of the study, symptoms compatible with COVID-19.
Brivio et al., 2021 [79]	– Italy<br>– Cross sectional<br>– N. 1,195, mean age = 40 y (SD = 14.948)	– Participants' Information-Seeking Behaviour (ISB)<br>– Impact of Event Scale Revised (IES_R)<br>– Generalized Anxiety Disorder scale (GAD-7)	– During the pandemic, the Italian population suffered a high level of distress (GAD-7 m = 6.89, s.d. = 5.08; IER-R mean score = 27.86, s.d. 17.46), respectively indicating mild presence of anxiety symptoms, and high levels of PTSD symptoms.<br>– Women scored significantly higher than men, both for anxiety

			symptoms and all dimensions of PTSD symptoms, avoidance, and hyperarousal.<br>– People from Generations Y and Z show to be at higher risk of developing PTSD and GAD symptoms.
Pieh et al., 2021 [80]	– UK<br>– Cross-sectional<br>– N. 1,006, 18-65+ y	– WHOQOL-BREF<br>– WHO-5 Well-Being Index<br>– Perceived Stress Scale (PSS-10)<br>– Patient Health Questionnaire (PHQ-9)<br>– Generalized Anxiety Disorder-7 (GAD-7)<br>– Insomnia Severity Index (ISI)<br>– Questionnaire about physical activity	– Younger age (< 35 y), female sex, unemployment and low income were risk factors for poor mental health.
Pierce et al., 2021 [101]	– UK<br>– Longitudinal<br>– N. 19,763, 16-70+ y	– 12-item General Health Questionnaire (GHQ-12)<br>– Data on sociodemographic information, housing, pre-existing mental conditions, COVID-related adversities, local lockdown restrictions	– Mental health of most UK adults remained stable or returned to pre-pandemic levels.<br>– Around one in nine individuals had deteriorating or consistently poor mental health.<br>– People living in areas affected by lockdown, struggling financially, with pre-existing conditions, or infection with SARS-CoV-2 might benefit most from early intervention.
García-Fernández et al., 2021 [97]	– Spain<br>– Cross-sectional comparison<br>– N. 1,839 people with a mental disorder (MD), 18-84 y compared	– Data on sociodemographic variables, COVID-related symptoms, information received related to infection (insufficient/adequate/excessive),	– MD group showed a significantly higher mean (SD) in anxiety [24.7 (11.8) vs. 17 (10.3)], depression [7.9 (6.0) vs. 4.2 (4.2)], and acute stress

	to N. 1,638 people non-MD, 18-73 y	coverage of basic needs, presence of violence, increase in the use of legal or illegal drugs, and practice of regular physical exercise<br>– Hamilton Anxiety Rating Scale (HARS)<br>– Beck Depression Inventory (BDI)<br>– Acute Stress Disorder Inventory (ASDI)	[6.3 (3.2) vs. 4.4 (3.1)] scores than non-MD.<br>– The COVID-19 confirmed cases rate was higher in MD participants than in non-MD.<br>– Among the MD group: being a COVID-19 confirmed case, the lack of basic needs coverage, the presence of violence, the drug use, and the absence of physical exercise were associated with more severe depressive symptoms.
Beutel et al., 2021 [86]	– Germany<br>– Cohort<br>– N. 2,503, 14-95 y	– Patient Health Questionnaire-4 (PHQ-4)<br>– A validated item to assess loneliness<br>– Generalized Anxiety Disorder 2-item (GAD-2)<br>– Questionnaire on sex, age, and socio-demographic factors such as low income, unemployment, migration status, and partnership	– Scores of depression and anxiety symptoms increased from an average of 0.89 (SD = 1.21) and 0.77 (SD = 1.17) in 2018 to 1.14 (SD = 1.23) and 1.05 (SD = 1.31) in 2020.<br>– Loneliness did not increase (M = 1.35, SD = 0.68 in 2018; M = 1.38, SD = 0.78 in 2020), affecting about one in four participants to some degree.<br>– Younger participants and women were most likely to report depression, anxiety, and loneliness.<br>– Social inequality factors contributed to distress and loneliness.
Skoda et al., 2021 [95]	– Germany<br>– Cross-sectional<br>– N. 12,028, 18-85+ y	– Questionnaire on socio-demographic factors, existing mental illnesses, and chronic somatic diseases	– Generalized anxiety, depressive symptoms, and distress were significantly increased in participants suffering from

		– An item for COVID-19-related fear<br>– General Anxiety Disorder-7 (GAD-7)<br>– Patient Health Questionnaire-2 (PHQ-2)<br>– A distress thermometer	mental illness in comparison to healthy control.<br>– COVID-19-related fear was comparable between patients suffering from multiple chronic diseases and those suffering from mental illness.
Pan et al., 2021 [67]	– The Netherlands<br>– Longitudinal<br>– N. 1,181 people with depressive, anxiety, or obsessive-compulsive disorders, mean age = 55.7 (SD = 12.9)<br>– N. 336 people without mental disorders, mean age = 57.7 (SD = 14.4)	– DSM-IV-based Composite Interview Diagnostic Instrument<br>– Structured Clinical Interview for DSM-IV axis I disorders<br>– Quick Inventory of Depressive Symptoms (QIDS)<br>– Beck Anxiety Inventory (BAI)<br>– Penn State Worry Questionnaire (PSWQ)<br>– De Jong Gierveld Loneliness Scale (DJGLS)<br>– Questionnaire on the living situation during the COVID-19 pandemic, diagnosis with COVID-19, current treatment for mental health disorders or feeling in need of treatment, perceived impact of the COVID-19 pandemic on their emotional state and health behaviors, coping strategies	– People without depressive, anxiety or obsessive-compulsive disorders showed a greater increase in symptoms during the COVID-19 pandemic.<br>– Individuals with the greatest burden on their mental health tended to remain stable, although systematically worse than that of people without these disorders.
van der Velden et al., 2021 [94]	– The Netherlands<br>– Longitudinal<br>– N. 4,084, 18-65+ y	– Data from Longitudinal Internet studies for the Social Sciences (LISS) panel<br>– Mental Health Index or Inventory (MHI-59<br>– De Jong Gierveld Loneliness Scale<br>– Questionnaire on physical illness	– Significant lower prevalence of anxiety and depression symptoms after the outbreak (T4 - June 2020 = 15.3%) than before (T2 - November 2019 = 16.8%) and during the COVID-19 outbreak (T3 - March 2020 = 17.2%).

			– Prevalence of emotional loneliness increased significantly after the outbreak (T1 = 18.4%, T4 = 24.8%).<br>– The prevalence of symptoms increased significantly among those who became lonely during the pandemic (T2 = 17.9%, T4 = 26.3%).
Orfei et al., 2022 [100]	– Italy<br>– Cross-sectional<br>– N. 1,401, 24-66 y	– Emotional Reaction Questionnaire (ERQ)<br>– Positive Affect and Negative Affect Scale (PANAS)<br>– Impact of Event Scale-Revised (IES-R)<br>– General Health Questionnaire (GHQ-12)<br>– Depression, Anxiety and Stress Scale (DASS-21)<br>– Dutch Work Addiction Scale (DUWAS)	– During post-lockdown, mean levels of symptoms of depression, anxiety, and stress levels fell in the normal range.<br>– A minority of participants showed higher levels of persistent psychological maladjustment (5.4% of participants reported post-traumatic symptoms and 15% signs of psychological distress).<br>– Gender (female), age (lower), and suppression emotional strategy showed to be significant predictors of a worse psychological outcome and a greater proneness to workaholism.
Pedersen et al., 2022 [98]	– Denmark<br>– Repeated cross-sectional<br>– N. 8,261, 18-65+ y	– Cantril Ladder scale<br>– UCLA 3-Item Loneliness Scale (UCLA T-ILS)<br>– Anxiety Subscale of the Common Medical Disorder Questionnaire (CMDQ-ANX)<br>– Mental Health Scale based on the core questions developed by	– Women, young individuals (<34 y), and those with a mental and/or chronic illness demonstrated poorer mean time series than others.<br>– People with a pre-existing mental illness further had a less reactive mental health time series.

		investigators at Johns Hopkins University	
Winkler et al., 2021 [87]	– Czech Republic<br>– Longitudinal<br>– N. 3,306 (T0), 18-55 y<br>– N. 3,021 (T1)<br>– N. 3,000 (T2)	– Mini-International Neuropsychiatric Interview (MINI)	– The proportion of individuals experiencing at least one mental disorder was highest during the second wave of the pandemic (32.94%, 95% CI = 31.14%; 34.77%), compared to both the baseline in 2017 (20.02%, 95% CI = 18.64%; 21.39%), and the first wave (29.63%, 95% CI = 27.9%; 31.37%).<br>– Younger adults, students, those having lost a job or on forced leave, and those with only elementary education displayed a disproportionally high prevalence of mental disorders.
Piumatti et al., 2022 [78]	– Southern Switzerland<br>– Longitudinal<br>– N. 732, 20-64 y	– 21-item Depression, Anxiety and Stress Scale (DASS-21)	– Prevalence of moderate to severe depression increased from 7.5% in August 2020 to 12.5% in May 2021, anxiety increased from 4.8% to 8.1% and stress increased from 5.5% to 8.8% on average, every month participants experienced a 6% increase in the odds of falling into the moderate to severe classification for depression, anxiety, and stress.<br>– Women had a higher risk for anxiety and stress.<br>– Suffering from a chronic somatic disease increased the risk for depression, anxiety, and stress.
González-Sanguino et al., 2021 [81]	– Spain<br>– Longitudinal	– Patient Health Questionnaire 2 (PHQ-2)	– Depressive symptoms increased significantly throughout the

	– N. 3,480 (T0), 18-60+ y<br>– N. 1,041 (T1)<br>– N. 569 (T2)	– Generalized Anxiety Disorder Scale-2 (GAD-2)<br>– Post-traumatic Stress Disorder Checklist (PCL-C-2)<br>– Intersectional Day-to-Day Discrimination Index (InDI-D)<br>– UCLA Loneliness Scale (UCLA-3)<br>– Multidimensional Scale of Perceived Social Support (EMAS)<br>– Functional Assessment of Chronic Illness Therapy Spiritual Well-Being (FACIT-Sp12)<br>– Self-Compassion Scale (SCS)<br>– Four items for a sense of belonging to different work/study groups, friends, family, and neighborhood or community	confinement, decreasing at the last assessment but not dropping to previous levels.<br>– Perceived loneliness and low spiritual well-being were the main predictors of mental health, as well as the lower age for depression and the female gender for anxiety and PSTD.
Vancea and Apostol, 2021 [82]	– Romania<br>– Longitudinal<br>– N. 543 (T1), 18-65+ y<br>– N. 583 (T2)	– IPAT Anxiety Scale<br>– Beck's Depression Inventory (BDI)<br>– Dissociative Experiences Scale (DES)	– T1: 23.8%, 19.2%, and 32.6% reported being clinically anxious, clinically depressed, and showed clinical dissociation, respectively.<br>– Approximately one in two respondents suffered from either anxiety, depression, or dissociation (t1= 46.0% and t2= 46.7%) and reported being very affected, indicating a major negative impact of the pandemic (t1= 51.6% and t2= 58.7%).<br>– Women's mental health status was alarming, 6 months after the end of the lockdown, with 60% of them reporting a significant impact; approximately one in

			three women (27.3%) suffered from clinical anxiety, while one in five (19.4%) suffered from clinical depression.<br>– Binary logistic regressions indicated that age, education level, and previous traumatic events were significantly associated with clinical levels of anxiety and depression.
Chodkiewicz et al., 2021 [68]	– Poland<br>– Cross-sectional<br>– N. 618, 18-76 y	– Hospital Anxiety and Depression Scale (HADS)<br>– Perceived Stress Scale (PSS 10)<br>– MINI-COPE Questionnaire (Brief COPE Inventory)<br>– Alcohol Use Disorder Identification Test (AUDIT)<br>– Scale of Death Anxiety (SDA)<br>– Fear of COVID-19 Scale (FCV-19S)	– 202 respondents (32.69%) suffered from anxiety disorders.<br>– 123 respondents (23.14%) were indicative of depressive disorders.<br>– Mean results obtained by means of the Perceived Stress Scale (= 24.67%) were within the range of high scores (8 sten).<br>– 96 respondents (15.86%) consumed alcohol in a hazardous or harmful manner (result > 8 points).<br>– 23.62% reported having experienced suicidal thoughts.<br>– More women, younger people, and those with disorders prior to the onset of the pandemic were among those who manifested these disorders. They also used passive and avoidance stress coping strategies more frequently.
Gambin et al., 2021 [88]	– Poland<br>– Cross-sectional<br>– N. 1,115, 18-85 y	– Patient Health Questionnaire-9 (PHQ-9)<br>– Generalized Anxiety Disorder-7 (GAD-7)	– Youngest individuals (aged 18–29) displayed significantly higher levels of anxiety and depression symptoms than those in the 45–59

		– Scale of Perceived Health and Life Risk of COVID-19<br>– Social Support Scale<br>– Scale of Pandemic-Related Difficulties<br>– Questionnaire on health problems, income continuity and financial situation	and 60–85 age groups despite lower level of perceived health and life risk of COVID-19.<br>– Individuals in the age range of 30–44 demonstrated higher levels of anxiety and depression symptoms than the oldest group of individuals.<br>– Household relationship difficulties (difficult relationships at home, inability to stay alone increased number of daily duties, loneliness, a feeling of being abandoned) were among the most significant predictors of depressive and generalized anxiety symptoms in all age groups.
Ayuso-Mateos et al., 2021 [102]	– Spain<br>– Longitudinal<br>– N. 1,103, mean age = 54.82 y (SD = 16.35)	– Composite International Diagnostic Interview (CIDI)<br>– A single item for suicidal thought<br>– Global Physical Activity Questionnaire version 2 (GPAQ-2)<br>– OSLO3 Social Support scale (OSS-3)<br>– UCLA loneliness scale<br>– 12-item World Health Organization Disability Assessment Schedule (WHODAS 2.0)<br>– Brief Resilience Scale (BRS)	– Younger individuals (odds ratio (OR) = 0.97 per year older) and those feeling loneliness (OR = 1.96) during the lockdown were at an increased risk of developing depression during the confinement. Resilience showed a protective effect against the risk of depression (OR = 0.46) and suicidal ideation (OR = 0.33), whereas individuals perceiving social support were at a lower risk of developing suicidal thoughts.<br>– (OR = 0.35).
O'Connor et al., 2022 [91]	– UK<br>– Longitudinal<br>– N. 3,077, 18-65+	– Two items for suicidal history and an item for suicidal ideation in the past week	– Rates of suicidal ideation increased over time: 8.2% at wave 1, 9.2% at wave 2, 9.8% at wave 3.

		– Patient Health Questionnaire (PHQ-9)<br>– Generalized Anxiety Disorder (GAD-7)<br>– Short Defeat and Entrapment Scale (SDES)<br>– Entrapment Scale Short-form<br>– Short Warwick Edinburgh Mental Well-Being Scale (SWEMWBS)<br>– UCLA Loneliness 3-item Scale (UCLA T-ILS)	– Levels of anxiety decreased over time: 21% at wave 1, 18.6% at wave 2, 16.8% at wave 3.<br>– Levels of depression remained high (26.1%) but stable across the waves.<br>– Men and women reported similar levels of suicidal ideation.<br>– Women, young people (18–29 years), those from more socially disadvantaged backgrounds and those with pre-existing mental health problems have worse mental health outcomes (depression, anxiety) during the pandemic.

Table S3. Elderly.

Author<br>Year	Country<br>Design<br>N, Age	Main Mental Health Measures (totally or in part)	Main findings
Garcia-Portilla et al., 2021 [104]	– Spain<br>– Cross-sectional<br>– N. 1,690 ≥ 60 y compared with N. 13,363 < 60 y	– Depression, Anxiety, and Stress scale (DASS-21)<br>– Impact of Event Scale (IES)	– 52.6% of women and 34.3% of men were found to be probable cases of any emotional distress.<br>– In both sexes, the most common psychological response was avoidance behavior (34.7% and 23.8%, respectively), followed by depression (28.5 and 14.2%).<br>– Older women and men were considered probable cases of any emotional distress less often than younger ones (women: 52.6% vs. 72.3%; men: 34.3% vs. 50.6%).<br>– The results of the binary logistic regression showed that only depressive and stress responses are psychological factors associated with age group.
Arpino et al., 2020 [108]	– Italy, France, and Spain<br>– Longitudinal<br>– N. 4,207, mean age 64.43 y	– Questionnaire focused on changes in physical and non physical contact, perceived depressive feelings at the time of the survey and perceived changes in depressive feelings since the	– 44% felt depressed more than usual during lockdown, and more frequently were women.<br>– Non-physical contact, especially if intergenerational, reduced the

		start of the lockdown	impact of lockdown on depressive feelings.
Dziedzic et al., 2021 [105]	– Poland<br>– Cross-sectional<br>– N.221, mean age mean = 65.18 y (SD = 4.06)	– Hospital Anxiety and Depression Scale (HADS-M)<br>– Revised UCLA Loneliness Scale (R-UCLA)	– Depressive symptoms presented in 19.15% of the participants (higher among women) and<br>borderline states in 14.18%.<br>– Based on R-UCLA, a moderate and moderately high sense of loneliness was present in 58.83% of the participants.<br>– Increasing loneliness scores were accompanied by increasing anxiety levels, depressive symptoms, and irritability scores.
Ceccato et al., 2020 [114]	– Italy<br>– Cross-sectional<br>– N. 102, 65-85 y compared with N.102, 30–50 y and N.102 young 18–29 y	– Positive and negative affect Schedule—PANAS<br>– Fear of COVID-19 Questionnaire with eight items, referring to either self or loved ones' health<br>– Attitudes toward the COVID-19 Questionnaire with 11 items referring to specific perceptions, ideas, and expectations about the pandemic situation<br>– A single item for behavioral response to the emergency	– Older adults reported significantly lower scores than both young and middle adults in emotional response to the emergency.<br>– Older adults were less worried about infection than both young and middle adults.<br>– Older adults were more confident about COVID-related information received, more favorable toward the restrictive measures, and perceived lower underestimation of the

			emergency compared to the other age groups.
Mayerl et al., 2021 [110]	– Austria<br>– Longitudinal<br>– N. 557, 60-89 y (wave 1)<br>– N. 463 (wave 2)	– Questionnaire on COVID-19-related social restrictions, feelings of loneliness, and symptoms of mental distress<br>– UCLA three-item loneliness scale (UCLA T-ILS)<br>– Brief Symptom Inventory (BSI)	– On wave 1, both social restrictions and loneliness were positively associated with depressive and anxiety symptoms. On the second wave, loneliness showed a positive correlation to both the depressive and anxiety symptoms and the social restrictions.<br>– More perceived social restrictions at wave 1 predicted more loneliness at wave 2, and vice versa, higher levels of loneliness at wave 1 predicted more perceived social restrictions at wave 2.<br>– More perceived social restrictions at wave 1 predicted higher levels of loneliness at the same point in time, which in turn predicted more depressive and anxiety symptoms at wave 2.<br>– No direct effect of social restrictions on depressive and anxiety symptoms at wave 1 and wave 2 was found.

Hansen et al., 2021 [111]	– Norway<br>– N. 10,740, 19-92 y (N. 1,053 65-74 y; N. 334 ≥75 y)	– UCLA Loneliness Scale Version 3 (UCLA LS3)<br>– Hopkins Symptom Checklist (HSCL-5)<br>– Oslo Support Scale (OSS-3)	– Women express loneliness in a U-shaped manner, across age in cross-sectional analysis, with the lowest rates in those aged 45–74 years.<br>– Among men, loneliness decreases with age in cross-sectional analysis<br>– Factors predicting significantly stronger decreases in loneliness were: age <75 y (for women only), being partnered, low social support, and high psychological distress.
Taylor et al., 2021 [109]	– Scotland<br>– Longitudinal<br>– N.190, > 80 y	– Data from LBC1936 study, a longitudinal study principally investigating non-pathological cognitive and brain ageing<br>– Moray House Test No.12 (MHT)<br>– Mini-mental state examination (MMSE)<br>– Rapid Estimate of Adult Literacy in Medicine (REALM)<br>– Test of Functional Health Literacy in Adults (TOFHLA)<br>– Newest Vital Sign (NVS)<br>– Townsend Disability Scale	– Modest declines in self-reported physical and mental health.<br>– Loneliness associated with living alone (OR = 0.15, 95%CI 0.07–0.31) and greater anxiety symptoms.

		– Hospital Anxiety and Depression scale (HADS-M)<br>– IPIP Big Five Personality Test<br>– Questionnaire on experience of COVID-19; knowledge and adherence to guidance; impact on day-to-day living; social contact; self-reported physical and mental health and loneliness; lifestyle	
Maggi et al., 2021 [107]	– Italy<br>– Longitudinal<br>– T0 n. 334 (aged ≥ 60 y), T1 n.50	– Perceived Memory and Attentional Failures Questionnaire (PerMAFaQ)<br>– Patient Health Questionnaire-9 (PHQ-9)<br>– Generalized Anxiety Disorder scale (GAD-7)<br>– DSM-5 Level 2-Anger-Adult measure (DSM 5-Anger)<br>– Brief Resilience Scale (BRS)<br>– Coping Scale<br>– Impact of Event Scale-Revised (IES-R)	– At T0 about 45% of the participants experienced depression, anxiety, or anger.<br>– More fear of getting infected was related to more severe depression, anxiety, and anger, but resilience was found to mediate these relationships.<br>– No significant difference was observed in mental health scores between T0 and T1.<br>– Post-traumatic stress disorder, evaluated by the IES-R, was subclinical or absent for 8% of respondents, whereas for 72% it was mild, for 16% it was moderate, and for 4% it was severe.
Giebel et al., 2020 [113]	– UK	– Index of Multiple Deprivation	– The group with the highest proportion scoring above the cut

	– Longitudinal<br>– N.61 people with dementia 45-88 y, n. 285 unpaid carers 22-95 y, and n. 223 older adults 65-90 y	(IMD)<br>– Short Warwick-Edinburgh Mental Well-Being Scale (SWEMWBS)<br>– Generalised Anxiety Disorder 7 (GAD-7)<br>– Personal Health Questionnaire 9 (PHQ-9)	off for both anxiety (33%) and depression (48%) were those living with dementia. In contrast, fewer older adults achieved caseness (5% anxiety and 5% depression). Amongst carers, proportions of anxiety and depression were higher amongst current carers (28%, 20%) than former ones (14%, 11%).<br>– For people living with dementia (PLWD), variation in social support hours was significantly related only to higher GAD-7 scores<br>– For carers, variation in social support hours was significantly related to the reduced scores on the SWEMWBS.<br>– For older adults, hours were significantly related to reduced scores on the SWEMWBS and higher scores on the GAD-7.
García-Prado et al., 2022 [112]	– 17 European countries<br>– Review<br>– N. 41,792, 50-75+ y	– Data from the Survey of Health, Ageing and Retirement in Europe (SHARE COVID-19 questionnaire) and from the Oxford COVID-19 Government Response Tracker for 17 countries	– Lockdown restrictions imposed during COVID-19 pandemic have worsened the mental health of senior and older Europeans, with estimated causal effects that amount to 5

			percentage points for insomnia, 7.2 percentage points for anxiety and 5.1 percentage points for depression.<br>– This effect was stronger for women and those aged between 50 and 65.
Zaninotto et al., 2022 [106]	– UK<br>– Longitudinal<br>– N.5,146, mean age 67.7 y (SD = 10.6)	– Centre for Epidemiological Studies Depression scale (CES-D-8)<br>– Generalized Anxiety Disorder scale (GAD-7)<br>– Control, Autonomy, Self-Realization and Pleasure (CASP-19)<br>– 3-item UCLA Loneliness Scale (UCLA T-ILS)	– Prevalence of clinically significant depressive symptoms increased from 12.5% before the COVID-19 pandemic to 22.6% in June and July 2020, with a further rise to 28.5% in November and December 2020, accompanied by slight increases in the mean total scores of loneliness and poor quality of life.<br>– Prevalence of anxiety rose from 9.4% to 10.9% from June and July 2020 to November and December 2020.<br>– Women and non-partnered people experienced worse changes in mental health.<br>– Participants with less wealth had lower levels of mental health than those in the highest wealth group, before and during the COVID-19 pandemic.

			Nevertheless, people with higher wealth experienced more negative changes in quality of life and loneliness throughout the COVID-19 pandemic.
